# Home-Based Hospice Care Reduces End-of-Life Expenditure in Taiwan: A Population-Based Study: Erratum

**DOI:** 10.1097/01.md.0000499792.63470.e6

**Published:** 2015-10-30

In the article “Home-Based Hospice Care Reduces End-of-Life Expenditure in Taiwan: A Population-Based Study”,^1^ which appeared in Volume 94, Issue 38 of *Medicine*, two *P* values in Table 1 were aligned in the incorrect column. The article has since been corrected online.
